# Evaluation of *Brevibacillus brevis* as a potential plant growth promoting rhizobacteria for cotton (*Gossypium hirsutum*) crop

**DOI:** 10.1186/s40064-016-2584-8

**Published:** 2016-06-30

**Authors:** Vibha Nehra, Baljeet Singh Saharan, Madhu Choudhary

**Affiliations:** Department of Microbiology, Kurukshetra University, Kurukshetra, Haryana 136 119 India; Division of Soil and Crop Management, Central Soil Salinity Research Institute, Karnal, Haryana 132 001 India

**Keywords:** *Brevibacillus brevis*, PGPR, Thermotolerant, Seed germination assay

## Abstract

The present investigation was undertaken to isolate, screen and evaluate a selected promising PGPR *Brevibacillus brevis* on cotton crop. Out of 156 bacterial isolates one of the most promising isolate was analyzed for the various PGP traits. A seed germination analysis was conducted with cotton seeds to evaluate the potential of the isolate to promote plant growth. The bacterial isolate was checked for its growth and survival at high temperatures. The isolate was also analyzed for the PGP traits exhibited after the heat treatment. To identify the isolate morphological, biochemical and molecular characterization was performed. The isolate was found positive for many of the PGP attributes like IAA, ARA, anti-fungal activity and ammonia production. Effect of seed bacterization on various plant growth parameters was used as an indicator. The isolate showed significant growth and exhibited various PGP traits at high temperature making it suitable as an inoculant for cotton crop. Isolate was identified as *Brevibacillus brevis* [SVC(II)14] based on phenotypic as well as genotypic attributes and after conducting this research we propose that the *B. brevis* which is reported for the first time for its PGP potential in cotton, exerts its beneficial effects on cotton crop through combined modes of actions.

## Background

Agriculture is the major sector for economic development of third world countries. To improve crop yield in an integrated plant nutrient management system the use of different biological approaches is becoming popular as an important additive to chemical fertilizers. One promising method to reduce the use of chemical fertilizers is the application of plant growth-promoting rhizobacteria (PGPR) as microbial inoculants in agriculture. PGPR has a potential role in developing sustainable crop production systems (Zahid et al. 2015). PGPR inhabit plant roots and exert a positive effect ranging from direct influence mechanisms to an indirect effect. PGPR can support the health of plants by improving soil fertility, nutrient availability and its uptake (Saharan and Nehra 2011; Majeed et al. 2015; Nehra and Choudhary 2015). Today the researchers are being able to use PGPR successfully for the field experiments as they have been potentially recognized for stimulating and increasing plant growth and crop yield. Several studies recently demonstrated that the combined use of PGPR as a biofertilizers and reduced amounts of chemical fertilizers applied could sustain soil fertility and crop yield (Bhardwaj et al. 2014). This integrated fertilization has been regarded as a promising method for the rational use of fertilizers to make agriculture more sustainable and productive. With the aim of enhancing the plant productivity a variety of bacteria are being used nowadays (Ullah and Bano 2015; Naqqash et al. 2016).

Increased growth and yields of many crops like maize, wheat and sweet potato (Calvo et al. 2010; Zahid et al. 2015; Majeed et al. 2015) by the use of PGPR have been reported worldwide. PGPR when used as inoculants play a key role in the biogeochemical cycles and have a great potential for use in agriculture and environmental protection (Zhou et al. 2015) by reducing the chemical fertilizer rate. However, in agriculture the use of microbial inoculants remain very low despite of using natural microbial resources to improve plant growth and health.

Cotton is an immensely important crop for the sustainable economy of the world and livelihood of the farming community. Nearly 75 % of the total raw material needed in the textile industry is being supplied by the cotton crop. There is a huge beneficial effect of nitrogen application on cotton crop as it helps in increasing the growth, yield and quality of the cotton (Narayanan et al. 1974). Thus, this enhanced plant growth can be achieved by supplying nitrogen by co-inoculating cotton plant with the diazotrophic PGPR. The biggest problem encountered with cotton is that it is highly susceptible to insects or plant pathogens which have a high impact on the cotton production. It is estimated that cotton consumes about 50 % of the total insecticides/pesticides used in the country. The inevitable use of pesticides increases the financial burden to the farmers as well as creates health and environmental risks (Makita 2012). Rhizospheric microbes suppress plant pathogens and may be considered as alternative to chemical pesticides. Production of toxins, antibiotics, hydrogen cyanide (HCN) and hydrolytic enzymes (lipases, proteases, chitinases) that degrade virulence factors or pathogen cell-wall components are some of the mechanisms by which the rhizospheric microbes directly inhibits the pathogen growth (Pereg and McMillan 2015). Many researchers have reported various bacteria which are found to increase growth and productivity of cotton crop like *P. Putida* (Yao et al. 2010), *P. alcaligenes* and *B. amyloliquefaciens* (Egamberdiyeva 2003), *P. extremorientalis* (Egamberdiyeva and Jabborova 2013), *Pseudomonas aeruginosa* and *Bacillus fusiformis* (Yasmin et al. 2013). The present study reports the isolation and identification of multi-trait *Brevibacillus* sp. having nitrogen fixing ability as well as other plant growth promoting activities. Its effect on the growth of cotton (*Gossypium hirsutum*) was also evaluated. As far as literature surveyed, this is the first report on *Brevibacillus* sp. that elicits plant growth promotion on cotton plant.

## Results and discussion

### Isolation of bacteria

A number of bacterial colonies were obtained on King’s B agar medium (King et al. 1954), Pikovskaya Agar Medium (Pikovskaya 1948), Jensen’s Agar Medium (Jenson 1954) and Nutrient Agar Medium. Colonies which had varied morphology and colony characteristics were picked (151) for their screening. All isolates were screened for the various PGP traits exhibited by them. Isolate no. SVC(II)14 showed best results in this primary screening over other isolates (unpublished data of Ph.D. thesis of Vibha Nehra).

### Plant growth promoting traits of test isolate

#### Indole acetic acid (IAA) production

**Fig. 1:**
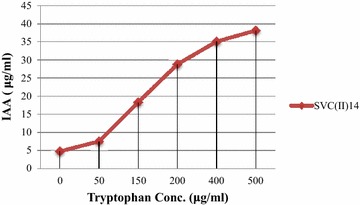
Effect of tryptophan concentration on IAA production

Screening results of PGP traits of isolate SVC(II)14 are depicted in Table 1. An important feature that can influence plant growth is the production of indolic compounds (phytohormones). These compounds stimulate root growth and increase root length, resulting in a larger root surface area that enables the plant to access more nutrients from the soil (Souza et al. 2013). The bacterial strain showed excellent IAA producing abilities and was able to produce IAA (4.74 μg ml^−1^). IAA production was found to be more pronounced in the presence of tryptophan and varying levels of IAA production were recorded. The isolate showed increase in the amount of IAA production (7.52, 18.31, 28.81, 35.09 and 38.16 μg ml^−1^) with the increased concentrations of l-tryptophan (50, 150, 200, 400 and 500 μg ml^−1^) shown in Fig. 1. Various researchers also reported increased auxin production in response to l-TRP application (Majeed et al. 2015; Iqbal et al. 2016). Ahmad et al. (2008) reported that several species of *Azotobacter*, *Bacillus and Pseudomonas* produced IAA up to 20, 10 and 25 µg ml^−1^, respectively. Iqbal et al. (2016) while carrying out the research under saline field conditions found that the IAA producing rhizobacteria significantly enhanced the growth, physiology and yield of Maize plants.

**Table 1:** PGP traits exhibited by the bacterial strain

Isolate	Phosphate solubilization	Ammonia production	HCN production	IAA production (μg ml^−1^)	ARA (nmol C_2_H_4_ mg^−1^ protein h^−1^)	Siderophore production	ACC deaminase activity	Anti fungal activity (mm)
*B. brevis*	−	++++	−	+++ (4.74)	+++ (10.25 ± 1.02)	−	−	+++ (20 ± 1.34)

++++, very good activity; +++, good activity; ++, significant activity; +, very low activity; −, negative results

The symbol ± refers to SE of the mean of three readings per treatment

#### Ammonia production

Another important PGP trait exhibited by the organism is ammonia production. Here the organism breaks down the complex nitrogenous material and releases ammonia in soil which is taken up by plant as a nutrient source. Accumulation of ammonia in the soil also creates the alkaline conditions which suppresses the growth of certain fungi (Jha et al. 2012). In the present study the isolate exhibited the very good ammonia production activity. From the rhizosphere of mangrove, rice and effluent contaminated soil 95 % of the isolates showed ammonia production which influenced the plant growth in an efficient way (Samuel and Muthukkaruppan 2011).

#### Acetylene reduction activity

Acetylene reduction activity (10.25 nmol C_2_H_4_ mg^−1^ protein h^−1^) was also exhibited by this selected isolate. It displayed its nitrogenase activity in terms of ARA. In the studies conducted by Badawi et al. (2011) it was found that uninoculated peanut plants showed nitrogenase activity in the range of 9.74–10.59 μmol C_2_H_4_ g^−1^ d.wt. of nodules h^−1^ but the rhizobial inoculation increased the rate of acetylene reduction by root nodules as they recorded 19.07 and 18.22 μmol C_2_H_4_ g^−1^ d.wt. nodules h^−1^, respectively, for the successive seasons. Hence microbial inoculations not only show PGPR activities itself but these can also enhance performance of plants.

#### Antifungal activity

The isolate SVC(II)14 was found positive for antifungal activity as it was showing zone of inhibition of 20 mm against test pathogen (*Macrophomina phaseolina*; Table 1). It was presumed that the isolate showed reduction in fungal growth and formation of inhibition zone by releasing antifungal substances and/or cell wall degrading enzymes into the culture medium. Various PGPR isolates were found to produce antagonistic activity against some phytopathogenic fungi such as *Fusarium oxysporum*, *Rhizoctonia solani* and *Sclerotium rolfsii* (Manivannan et al. 2012). While working on tomato seedlings Walia et al. (2013) isolated some bacterial strains from rhizosphere soil which were found to show broad spectrum antifungal activity against *F. oxysporum*, *R. solani* and *S. sclerotiorum*.

However, production of HCN, ACC deaminase activity, Phosphate solubilization and siderophore formation was not detected in SVC(II)14 (Table 1).

#### Seed germination

**Fig. 2:**
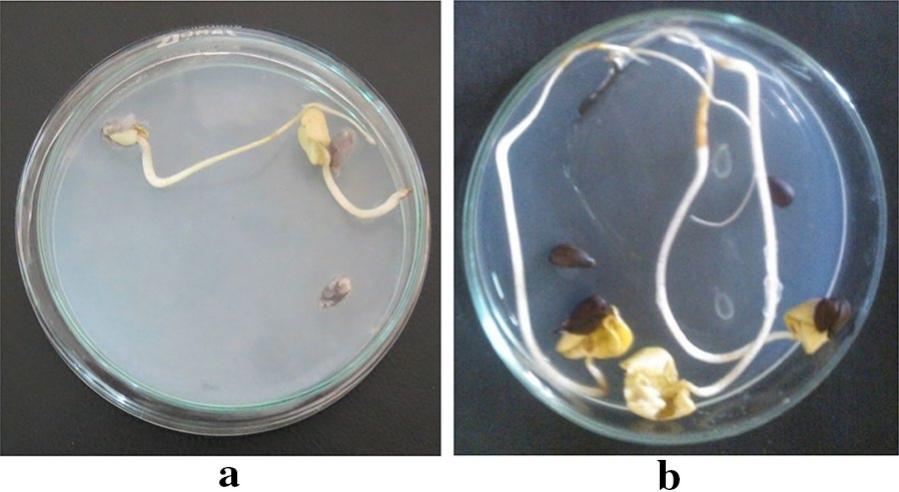
Effect of bacterial isolate SVC(II)14 treatment on seed germination of cotton (*Gossypium hirsutum*) after 7 days. **a** Control (without bacterial isolate). **b** Seeds treated with SVC(II)14

Seedling establishment, crop growth and productivity are greatly influenced by seed vigour and viability. Our studies were concentrated on studying the effect of screened PGPR isolates on seed germination (Table 2). Germination percentage and speed were increased by the effect of inoculation. Vigour index was increased to double of the control. Shoot (hypocotyl) length and root length were recorded respectively 75 and 84 % more than control due to the seed treatment by SVC(II)14. It was observed that root growth was affected more than shoot growth as it was evident from their fresh and dry weights. Fresh weights of shoot and root were increased by 82 and 129 % whereas dry weights were increased by 103 and 135 % respectively. Seedling emergence and seedling vigor due to the bacterial inoculation were considered significant on the basis of increase in root and shoot length (Fig. 2). In our study inoculation of cotton seeds with PGPR isolate had some growth promoting effects on shoot length and root length of plant. It was also observed by other researchers that PGPR increases root and shoot (hypocotyl) lengths. Increased synthesis of hormones like gibberellins could be the reason behind such findings, which resulted in an increased availability of starch assimilation. Gibberellins in the seed embryo are believed to signal starch hydrolysis through inducing the synthesis of the enzyme α-amylase. α-Amylase then hydrolyses starch, which is abundant in many seeds, into glucose that can be used in cellular respiration to produce energy for the seed embryo.

**Table 2:** Effect of bacterial culture SVC(II)14 on seed germination

Isolate	Germination (%)	Germination speed	Vigour index	Shoot length (cm)	Root length (cm)	Fresh shoot wt (mg seed^−1^)	Fresh root wt (mg seed^−1^)	Dry shoot wt (mg seed^−1^)	Dry root wt (mg seed^−1^)
Control	89 ± 0.34	8.52 ± 0.1	388 ± 48	1.03 ± 0.1	3.34 ± 1.5	125 ± 1.1	105.3 ± 4.3	11.6 ± 2.0	10.01 ± 0.2
SVC(II)14	96.6 ± 0.57	10.82 ± 0.3	807 ± 94	2.21 ± 0.2	6.14 ± 0.1	230 ± 11.1	341.2 ± 5.6	23.6 ± 1.5	29.58 ± 3

The symbol ± refers to SE of the mean of three readings per treatment

#### Effect of temperature on bacterial growth

**Fig. 3:**
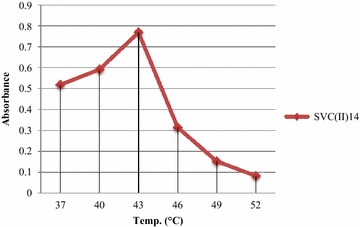
Effect of temperature on the growth of SVC(II)14

The bacterial culture was inoculated in the Nutrient broth and given heat shock treatment by keeping them at various temperatures (37, 40, 43, 46, 49, 52 °C) for a week under static conditions. The bacterial growth was measured through spectrophotometer by taking readings at 600 nm. The isolate showed a lot of variation in their growth with increasing temperature. It was found that isolate SVC(II)14 could survive at 52 °C. The isolate showed 0.519 and 0.592 absorbance at 37 and 40 °C, respectively. With the increase in the temperature 46, 49 and 52 °C, the absorbance of the isolate decreased to 0.314, 0.152 and 0.082, respectively. Maximum absorbance of 0.769 was noticed at 43 °C (Fig. 3). These results indicates that SVC(II)14 can tolerate high temperatures as the cotton crop is taken in areas where soil temperature shoots up to 47 °C so for proper functioning of bacterial isolate it should be thermotolerant.

#### Effect of temperature on PGP traits

After giving heat shock treatment to the isolate and checking its growth with increasing temperature the isolate was checked for the various PGP traits exhibited by it at higher temperatures i.e. 43 and 46 °C. Significant production of IAA (1.4 μg ml^−1^) was shown by the selected thermotolerant isolate at 43 °C but it did not show any activity at 46 °C. It shows that the IAA producing ability of the isolate decreases with the increase in the temperature, similar results were also reported by Sudha et al. (2012). At 46 °C the ARA activity was found to be 1.92 nmol C_2_H_4_ mg^−1^ protein h^−1^. But when the heat treatment temperature was reduced to 43 °C the activity was found to be 3.21 nmol C_2_H_4_ mg^−1^ protein h^−1^. In the present study when the isolate was checked for ammonia production at higher temperatures the isolate showed positive results for the activity at both 43 and 46 °C but the ammonia produced was more pronounced at 43 °C. At 43 and 46 °C the isolate did not show any anti-fungal activity (Table 3) but when the activity was checked at 40 °C the isolate was found to show positive results with the zone of inhibition of 10 mm. Bhosale et al. (2013) also obsereved similar trend, while working on *Azotobacter vinelandii* it was found that the temperature conditions affects antifungal metabolite production of the bacteria. Similarly Bilkay et al. (2010) while working on a fungus *Aspergillus niger* found that the antifungal activity decreases with increase in temperature. These results indicate that at higher temperatures the tested isolates could exhibit more than two or three PGP traits, which may promote plant growth directly, indirectly or synergistically.

**Table 3:** PGP activity indices of tested thermotolerant isolate after 4 days of incubation

Activity	Temperature		
	43 °C	46 °C	
IAA production (μg ml^−1^)	1.4 ± 1.03	−	
ARA (nmol mmg^−1^ protein h^−1^)	3.21 ± 2.21	1.92 ± 1.98	
Ammonia production	++	+	
Anti fungal activity (mm)	−	−	10 ± 0.47 (at 40 °C)

The symbol ± refers to SE of the mean of three readings per treatment

#### Morphological and biochemical analysis of the strain

The isolate SVC(II)14 strain was gram positive, motile rod with endospore formation. It was punctiform with raised elevation and entire margin. The colony was creamish white in color with rough surface. It showed positive results for catalase test and nitrate reduction test. It produced negative results for starch hydrolysis, oxidase test, indole test and VP test. Out of all it could ferment only galactose and not glucose and sucrose. The isolate could utilize fructose, galactose, mannose, salicin and rhamnose as carbohydrates. According to Bergey’s Manual of Determinative Bacteriology SVC(II)14 matched well with genus *Brevibacillus* (Table 4).

**Table 4:** Biochemical characterization of the isolates

Test	SVC(II)14	Test	SVC(II)14
Catalase test	+	Fructose	+
Oxidase test	−	Lactose	−
Indole test	−	Galactose	+
VP test	−	Trehalose	−
Nitrate reduction test	+	Sucrose	+
Starch hydrolysis	−	Mannose	+
Urease test	−	Glycerol	−
Carbohydrate fermentation test		Salicin	+
Glucose	−	Mannitol	−
Sucrose	−	Rhamnose	+
Galactose	A		

#### Phenotypic microarray analysis of the strain

The isolate was analyzed phenotypically by using Biolog system. After reduction of the tetrazolium dye the purple color is formed for the positive reaction, blue color if the result is not clear and no color indicates the negative results (Table 5). The isolate was identified up to 99 % as *Brevibacillus* sp. by the Omnilog instrument.

**Table 5:** PM analysis of the isolate SVC(II)14 by OmniLog

Substance(s)	Result(s)	Substance(s)	Result(s)	Substance(s)	Result(s)
d-Raffinose	−	Glycerol	+	Stachyose	+
α-d-Glucose	−	l-Aspartic acid	+	*N*-Acetyl neuraminic acid	+
d-Sorbitol	+	d-Glucuronic acid	+	Bromo succinic acid	+
Gelatin	−	Citric acid	+	Inosine	+
Pectin	+	α-Keto-butyric acid	+	d-Serine	+
*p*-Hydroxy-phenylacetic acid	+	Gentiobiose	+	l-Serine	+
Tween 40	−	*N*-Acetyl-d-glucosamine	+	d-Saccharic acid	+
Dextrin	+	d-Fructose	−	Bromo succinic acid	+
α-d-Lactose	+	d-Glucose-6-PO_4_	+	Formic acid	+
d-Mannose	−	l-Glutamic acid	+	1 % NaCl	+
d-Mannitol	+	Glucuronamide	+	1 % sodium lactate	+
Glycyl-l-proline	+	α-ketoGlutaric acid	+	Troleandomycin	−
d-Galacturonic acid	−	Acetoacetic acid	+	Lincomycin	+
Methyl pyruvate	+	Sucrose	+	Vancomycin	−
*g*-Amino butyric acid	−	N-Acetyl-β-d-mannosamine	+	Nalidixic acid	−
d-Maltose	−	l-Fucose	−	Aztreonam	−
d-Melibiose	−	Formic acid	+	ph6	+
d-Fructose	+	d-Serine	+	4 % NaCl	−
d-Arabitol	+	d-Fructose-6-PO_4_	+	Fusidic acid	−
l-Galactonic acid	+	l-Histidine	+	Rifamycin SV	−
d-Lactic acid methyl ester	+	Mucic acid	+	Guanidine HCl	+
α-Hydroxy-butyric acid	−	d-Malic acid	+	Tetrazolium violet	−
d-Trehalose	+	Propionic acid	+	Lithium chloride	−
B-Methyl-d-glucoside	+	l-Serine	−	Sodium butyrate	−
d-Galactose	−	d-Turanose	−	Ph5	−
Myo-Inositol	+	*N*-Acetyl-d-galactosamine	+	8 % NaCl	−
l-Arginine	+	l-Rhamnose	+	d-Serine	+
d-Gluconic acid	+	d-Aspartic acid	+	Minocycline	−
l-Lactic acid	+	l-Pyroglutamic acid	+	Niaproofu	−
α-Hydroxy-d	+	d-Saccharic acid	+	Tetrazolium blue	−
d-Cellobiose	+	Quinic acid	+	Potassium tellurite	+
d-Salicin	+	l-Malic acid	−	Sodium bromate	+/−
3-Methyl glucose	−	Acetic acid	+		

± delayed result, +/− unclear results

#### Identification

**Fig. 4:**
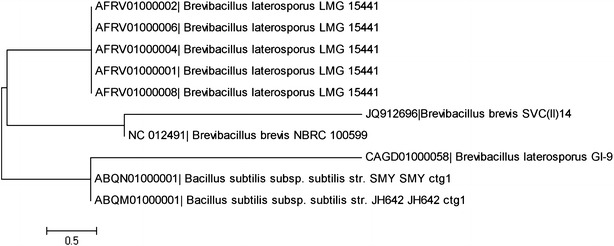
Phylogenetic relationship of SVC(II)14 based on 16S rDNA sequence. The plant growth promoting bacterial isolate SVC(II)14 with other closely related bacterial strains resulted from Blastn search

On the basis of biochemical characteristics and phenotypic microarray analysis the isolate SVC(II)14 was found to belong to *Brevibacillus* genus. For the molecular identification both strands of the DNA of isolate were sequenced. Amplified gene sequence length was found of 957 bp. The sequenced PCR products of the bacterial isolate was matched with the available sequences in the GenBank database and showed 99 % similarity with the brevis species (Fig. 4).

#### Role of Brevibacillus brevis in plant growth

*Brevibacillusbrevis* is considered PGPR and is widespread in the soil and sediment, and it has been widely used in agriculture and environmental remediation because of its multiple potential functions. Many researchers have worked with this bacterium as a PGPR on other crops. While working on plant Tomato, Girish and Umesha (2005) discovered that the *B. brevis* IPC11along with other strains provided maximum protection from the disease bacterial canker of tomato. Chandel et al. (2010) found that *B. Brevis* is a potential biological control agent for reducing the impact of *F. oxysporum* f.sp. *lycopersici* on tomato. Working on the same bacterium, Vivas et al. (2006) suggested that *B. brevis* inoculation improved the mycorrhizal benefit in nutrients uptake and in decreasing Ni toxicity in Clover (*Trifolium repens*) and it may be used as a tool to enhance plant performance in soil contaminated with Ni. Haggag et al. (2013) suggested the encouraged use of *Brevibacillusbrevis* and *Bacillus polymyxa* on a large scale for biocontrol of pre and post harvest strawberry from Gray Mould Disease. We claim to be working on *Brevibacillus brevis* as a potent PGPR in cotton crop for the first time. Thus our results should encourage the use of *Bacillus brevis* on a large scale for enhancing the growth and productivity of cotton crop.

#### Accession number

The sequence was submitted to the National Centre for Biotechnology Information (NCBI) and is available under the Genebank accession no. JQ912696.

## Conclusion

In our study *Brevibacillus brevis* SVC(II)14 exerts its beneficial effects on cotton plants through combined modes of actions, including exhibiting various PGP traits like phosphate solubilization, IAA production, Acetylene Reduction and anti-fungal activity. *B. brevis* SVC(II)14 also accelerating the growth and development of the cotton plant, enhancing root function as evident by seed germination assay results. The capability to survive higher temperatures makes our isolate a suitable inoculant for the cotton crop as it can sustain these harsh environments. Its ability to exhibit various PGP traits at higher temperature shows that the isolate may prove effective as an inoculant. The additional feature of motility the isolated bacterium leads to suppose that they can survive better in the environment.

## Methods

The research experiments were carried out in the Bioresource Technology Lab, Kurukshetra University, Kurukshetra. PGPR was isolated from rhizospheric soil of cotton plants. These bacterial isolates were characterized for their morphological and biochemical characters. The isolates were also characterized for the PGP traits exhibited by them. Seed germination assay was conducted for the evaluation of the effects of PGPR on cotton seed germination and growth of seedlings in controlled conditions.

### Sample collection

The cultures used in the present study were isolated from the set of ten soil samples collected from the rhizospheric soil of the cotton (*Gossypium hirsutum*) growing fields of Haryana (INDIA). The plants were uprooted at the first square stage and the soil attached to the roots of the plants was taken aseptically. The samples were then properly stored at 4 °C in the laboratory up to a week in sterilized polyethylene bags. Collected soil samples were then used for the isolation of bacteria using serial dilution method. The soil suspension was spread on various media which include King’s B agar medium (King et al. 1954), Pikovskaya Agar Medium (Pikovskaya 1948), Jensen’s Agar Medium (Jenson 1954) and Nutrient Agar Medium. Individual colonies of bacteria, which varied in morphology (shape, size and colour) were picked up using a sterile inoculating wire loop and subcultured to purify by repetitive streaking on NA plates. The purity of each bacterial isolate was checked under the microscope using standard staining methods. The purified isolates were maintained on NA media and kept at 4 °C and subcultured at an interval of every 4 weeks. The purified colonies of the isolates were preserved in 10 % glycerol at −20 °C.

### Primary screening

#### Detection of plant growth promoting traits of the isolates

All the isolates were screened for the expression of plant growth promoting attributes. In vitro IAA production was estimated colorimetrically (with and without tryptophan in the medium). The Salkowski reagent was used to estimate IAA production and quantified spectrophotometrically by measuring the intensity of pink colour at 530 nm. The standard calibration curve was prepared by using standard IAA stock solution (0–100 μg ml^−1^) which was prepared in 50 % ethanol (Gordon and Weber 1951). Pikovskaya medium containing tricalcium phosphate was used for the qualitative assay for P solubilization (Pikovskaya 1948) and the isolates showing the clear halo zone around the culture spot, after incubation for 48 h 30 °C, indicated the P solubilization capacity of the isolate. For qualitative analysis Pikovskaya’s broth was inoculated with PGPR isolates and incubated at 28 ± 2 °C on a rotary shaker. After 6–7 days of incubation water soluble Phosphorous was determined in the supernatant by the method of Olsen et al. (1954). HCN production was evaluated by the qualitative method of Bakker and Schippers (1987). In this the isolates were streaked in the sterilized King’s B medium amended with 4.4 g l^−1^ of glycine. Whatman (No. 1) filter paper disc (9 cm in diameter) soaked in picric acid (0.05 % solution in 2 % Sodium Carbonate) was placed in the lid of each Petri plate. Colour change of the filter paper from deep yellow to orange and finally to dark brown show positive results of HCN production. Ammonia production was detected by growing the isolates in peptone broth for 72 h and then adding 1 ml of Nessler’s reagent to it. The isolates showing yellowish brown colour were found to exhibit positive results (Dye 1962). Chrome Azurole’s (CAS) agar media was used for the qualitative assay of siderophore production (Schwyn and Neilands 1987) and the isolates which showed a yellow orange halo around the colony were taken as positive results. The activity of 1-aminocyclopropane-1-carboxylate (ACC) deaminase enzyme in the induced bacteria was quantified by measuring α-ketobutyrateamount produced by the enzymatic cleavage of ACC (Jacobson et al. 1994). The dual culture technique was used for accessing the antifungal activity against the test pathogen (*Macrophomina phaseolina*) on Potato Dextrose Agar (PDA) medium (Dennis and Webster 1971) and after 48 h the diameter of zone of inhibition was measured. Nitrogen fixing ability of the isolates was determined by Acetylene reduction assay in nitrogen free medium. The pure cultures of the isolates were inoculated on a semi-solid nitrogen-free medium (Rennie 1981) which was prepared by solution A and solution B with 1 % agar. Solutions A and B were mixed together after being autoclaved separately and after filter sterilization Biotin (5 μg l^−1^) and PABA (10 μg l^−1^) were also added to the above mixture. After inoculating the isolates on this nitrogen-free semi- solid medium they were incubated at 30 °C for 48 h under stationary conditions. After incubation, the air was replaced with acetylene (10 % v/v) and the tubes are again incubated at 30 °C for 6 h. Ethylene production was measured using a Gas chromatograph and the total cell pellet obtained after centrifugation was digested by adding 500 μl of 2 N NaOH and kept at 100 °C for 30 min. After cooling, equal amounts of 2 N HCl were added to neutralize the aliquot. The values of ARA expressed in terms of nmol C_2_H_4_ mg^−1^ protein h^−1^and the protein concentration of the cell suspension was estimated by the method of Lowry et al. (1951).

#### Secondary screening of the bacterial isolates

To evaluate the potential for plant growth promotion of the screened isolate, germination and seedling vigor tests were performed with cotton (*Gossypium hirusutm*) seedlings. The seeds of the variety Pf-6 were taken from Indian Agriculture Research Institute, Delhi. The seeds were surface-sterilized with 1 % sodium hypochlorite for 10 min. The seeds were inoculated by soaking in the log phase bacterial culture containing at least 10^6^ CFU ml^−1^ for 15 min. The seeds of the control treatments were soaked in sterile water. Soft agar plates (0.8 % sterile agar) were prepared and dipped seeds of each treatment with three replicates were placed on them at 28 °C for 4–5 days. After being incubated for 4–5 days, the germinated seeds were counted. Seed vigor index was calculated by multiplying germination (%) by seedling length (mm; Abdul Baki and Anderson 1973). After 6 days, seedlings were taken out from each Petri dish and their respective root and shoot lengths were measured. Seedling components were separated into root and shoot and measured separately for their fresh weight. These seedling parts were dried in an oven for 12 h at 45 °C and their dry weight was taken. The plant growth measurements i.e. shoot length, root length, wet shoot weight, wet root weight, dry shoot weight, dry root weight, vigor index and emergence index were noted in 7 day old seedlings.

### Characterization of potent isolates

#### Morphological characterization of isolates

Morphological features of the selected strain were determined which included: color-pigment, form, elevation, margin, and surface. The Gram reaction and endospore staining was performed as per standard procedures (Park et al. 2005). The motility of the bacteria was checked using hanging drop method.

#### Biochemical characterization

All the bacterial isolates were tested for their positive or negative response to different biochemical tests on the basis of which they were compared to different representative genera in Bergey’s Manual of Determinative Bacteriology (Holt et al. 1994). On the basis of these tests binary data was generated which was further used for dendrogram preparation and profiling of metabolic diversity of the representative genera. The various biochemical tests were performed including oxidase test, casein hydrolysis, starch hydrolysis, catalase test, urease test, nitrate reduction test (Cappuccino and Sherman 2008).

#### Phenotypic microarray analysis

Phenotypic microarray (PM) analysis was done at CIF, Delhi University, New Delhi. PM dissects and analyzes the species on the basis of cell’s ability to metabolize major classes of biochemicals and some important physiological properties such as pH, salt, lactic acid tolerance, chemical sensitivity and reducing power. The chemical reaction taking place between the bacteria and a tetrazolium dye is the base of PM Technology. If the medium in the well supports the bacterial growth, the tetrazoliumis reduced by the metabolizing cells and colour is produced, which over a period of 24 h can be measured with the OmniLog instrument. Both the inoculating medium and the concentrations of substrates are proprietary information (www.biolog.com). Further information on the compounds tested in the PM analysis can be found at the Biolog website.

#### DNA isolation, amplification and rDNA homology

To obtain the pure culture the isolate was raised in 5 ml nutrient broth for 18–24 h to obtain O.D. of 0.6 at 600 nm. The culture broth was microfuged for 2 min at 12,000 rpm to obtain the pellet (1.5 ml). The bacterial culture in the pellet form was sent to the Oscimum Link Biotech, Hyderabad for DNA isolation, PCR amplification and 16S rDNA sequencing. Total genomic DNA of the bacteria was isolated by using method of Charles and Nester (1993) with slight modification. The amount of DNA was estimated by spectrophotometer at 260 nm using the relationship that O.D. of 1.0 corresponds to 50 g ml^−1^. Quantification of the bacterial genomic DNA was done by agarose gel electrophoresis by analyzing their migration on 0.8 % agarose gel prepared in 0.5 M Tris–borate–EDTA (TBE) buffer.

Forward primer 27F (5′-AGAGTTTGATCCTGGCTCAG-3′) and Reverse primer 1492R (5′-GGTTACCTTGTTACGACTT-3′; Maatallah et al. 2002) used for amplification of 16S rDNA genes. In polymerase chain reaction the following programme was used for the amplification of 16S rDNA: The reaction was performed at 95 °C for 5 min, then 30 cycles reaction of 94 °C for 1 min, 55 °C for 1 min and 72 °C for 1 min followed by a final extension of 10 min at 72 °C.

PCR products were purified by dissolving 10–15 μl of the unpurified DNA sample in 50 μl of PCR cleanup solution and incubated at 55 °C for 15–20 min. The mixture was centrifuged at 12,000 rpm for 15 min and the supernatant was discarded. The DNA was further precipitated by the addition of 600 μl of 80 % ethanol and the supernatant was discarded after centrifugation. Finally the DNA pellet was dried and dissolved in 10–15 μl of Milli Q water.

The sequencing of the target gene was done using BigDye Chemistry, and performed as per the manufacturer’s protocols (Applied Biosystems 3730 xl DNA Analyzer) in Ocimum’s lab. Sequence data analysis was done by using Chromas Proand Sequencing Analysis software. To identify the isolate partial sequence of 16S rRNA gene were analysed using NCBI BLAST tool. The query sequence was multiple aligned against non-redundant (nr) nucleotide sequence database of NCBI using Mega5 program for phylogenetic tree preparation and comparison of the query sequence with other phylogenetically closed microbial relatives. The gene sequence was submitted to GenBank of NCBI.
